# Identification of Novel Bile Salt-Tolerant Genes in *Lactobacillus* Using Comparative Genomics and Its Application in the Rapid Screening of Tolerant Strains

**DOI:** 10.3390/microorganisms10122371

**Published:** 2022-11-30

**Authors:** Chunfei Chen, Leilei Yu, Fengwei Tian, Jianxin Zhao, Qixiao Zhai

**Affiliations:** 1 State Key Laboratory of Food Science and Technology, Jiangnan University, Wuxi 214122, China; 2 School of Food Science and Technology, Jiangnan University, Wuxi 214122, China

**Keywords:** bile salt tolerance, comparative genomics, *Lactobacillus*, malate dehydrogenase, rapid screening

## Abstract

Under bile salt treatment, strains display significant differences in their tolerance ability, suggesting the existence of diverse resistance mechanisms in *Lactobacillus*; however, the genes involved in this protective process are not fully understood. In this study, novel target genes associated with bile salt tolerance in *Lactobacillus* were identified using comparative genomics for PCR detection and the rapid screening of tolerant strains. The bile salt tolerance of 107 *lactobacilli* isolated from different origins was assessed, and 26 strains with comparatively large differences were selected for further comparative genomic analysis. Tolerant strains had 112 specific genes that were enriched in the phosphotransferase system, the two-component system, carbohydrate metabolism, and the ATP-binding cassette transporter. Six genes from *Lactobacillus* were cloned into the inducible lactobacillal expression vector pSIP403. Overexpression in the host strain increased its tolerance ability by 11.86–18.08%. The novel genes identified here can be used as targets to design primers for the rapid screening of bile salt-tolerant *lactobacilli*. Altogether, these results deepen our understanding of bile salt tolerance mechanisms in *Lactobacillus* and provide a basis for further rapid assessments of tolerant strains.

## 1. Introduction

Currently, commercially-available *lactobacilli* have specific health-promoting properties [1,2]. To survive gastrointestinal transport and successfully reach the colon in a feasible status, these bacteria must overcome serious challenges throughout the gastrointestinal tract, especially the presence of acid in the stomach and bile salt in the upper parts of the small intestine [3]. Most *lactobacilli* can survive in acidic conditions as low as pH 2–3 owing to their excellent ability to metabolize sugars to produce lactic acid [4]. Nevertheless, few studies have reported considerable variability in the response of *lactobacilli* to bile salt exposure [5]. A gastrointestinal stress evaluation of 184 *lactobacilli* revealed that 12% of the strains had a high growth capacity and 38% did not grow after 24 h of cultivation at a bile salt concentration of 1.5% [6]. Thus, an evident heterogeneity in the bile salt tolerance of *lactobacilli* was identified.

In addition to general physiological functions, bile acids are weak acids with detergent-like properties, and they are extremely poisonous for some intestinal microbes that inherently restrict the growth of bacteria [7]. Indeed, bile salt efflux, bile salt hydrolases (BSHs), and changes in the composition of bacterial membranes appear to be the most common mechanisms in *lactobacilli* to cope with the detrimental effects of bile salts [8]. Genes (*LSL_1310*, *LSL_1034*, *LSL_1371*, *murL*, *dacC*, *murE*, and *mleS*) associated with the autolysis and biosynthesis of peptidoglycan, a major component of the cell surface, are upregulated after bile salt stress [9,10]. Simultaneously, ClpP, Dps, GroEL, Hsp1, and Hsp3 are some of the general stress response proteins involved in repair and protection that are upregulated to counteract the oxidative stress caused by bile salts in *L. plantarum* [11]. In conclusion, bile salt tolerance is a multifactorial phenomenon, and the mechanisms by which *lactobacilli* survive bile salt-induced stress with associated genes remain to be elucidated.

To investigate the survival mechanism of *lactobacilli* under harsh conditions, transcriptomics and proteomics have been increasingly applied to reveal the key pathways of bile salt resistance [10,12,13]. Nevertheless, comparative genomics, which can normally be used to reveal species lifestyles, has rarely been applied in bile salt tolerance research. Based on the increasing number of publicly available whole genome sequences of strains, comparative genomic analysis has been applied to identify core genomes that are shared by all strains and accessory genomes that are shared by several but not all strains [14,15]. Similarly, comparative genomics was applied to reveal the core metabolic pathways and genetic basis of the properties of *lactobacilli*, suggesting the potential of applying this genomics to reveal the association between the phenotypes and genotypes of bile salt-resistant *lactobacilli* [16]. Studies on bile salt tolerance in *lactobacilli* have revealed the mechanisms in *L. rhamnosus* GG [12], *L. paracasei* L9 [10], *L. johnsonii* PF01 [17], and *L. fermentum* NCDC400 [18]. Notably, owing to the limited number of strains in these studies, the patterns underlying bile salt tolerance in *lactobacilli* have not been revealed. In addition, bile salt resistance-related genes have not been validated compared with other phenotypes, resulting in an absence of genes that could be used as targets for screening tolerant strains [19].

Here, a comparative genomic approach was applied to identify novel genes associated with bile salt tolerance in *lactobacilli*. Based on the phenotypic differences in 107 *lactobacilli*, the relationship between strain origin and bile salt tolerance was re-examined and the functional genes were re-predicted. The functional genes were overexpressed using a pSIP expression vector to validate their reliability as candidates for novel bile salt tolerance genes. These results provide a feasible basis for the rapid detection of bile salt-resistant *lactobacilli* by PCR amplification.

## 2. Materials and Methods

### 2.1. Bacterial Strains, Plasmids, and Growth Conditions

Detailed information on the strains is provided in Table S1. The study was carried out using 107 *lactobacilli*, including *L. fermentum*, *L. plantarum*, and *L. paracasei*. The strains were isolated from dairy products, fermented vegetables, and feces. The specific screening and isolation conditions were as described by Huang et al. [20]. *Lactobacilli* were cultured at 37 °C in a de Man–Rogosa–Sharpe (MRS) medium for three generations without agitation. *Escherichia coli* DH5α was used as the cloning host and was cultured at 37 °C in a Luria Bertani (LB) medium with shaking at 220 rpm (Table 1). When appropriate, 10 μg/mL or 500 μg/mL of erythromycin (Em) was added for *Lactobacillus* or *E. coli*, respectively.

### 2.2. Bile Salt Tolerance Evaluation

The bile salt tolerance of the strains was determined according to the method described by García-Ruiz et al. with slight modifications [21]. *L. rhamnosus* GG, a widely characterized and intensively investigated probiotic strain, was used as a reference control. Overnight cultures were inoculated (2% *v*/*v*) into a MRS medium with or without 1.2% bile salt (*w*/*v*) (Oxoid LP0055). The cultures were incubated for 12 h to measure the optical density at 600 nm (OD_600_) for the preliminary evaluation of bile salt tolerance. To obtain strains for subsequent validation experiments, tolerant and non-tolerant strains were partially selected for gradient bile salt tolerance (0%, 0.3%, 0.6%, 0.9%, 1.2%, 2%, 3%, 4%, 6%, 8%, and 10% bile salt), and the OD_600_ was measured as previously described. The growth rates of the strains are expressed as the percentage of OD_600_ under different bile salt concentrations relative to that of the control. All experiments were performed in triplicate.

### 2.3. Genome Sequencing and Annotation

The strains used in this study were transported on dry ice for whole-genome sequencing (Majorbio, Shanghai, China). The genome frame was constructed using Roche454 long-fragment double-end sequencing technology (paired-end), using Illumina short-fragment high-coverage sequencing (100×), mapping the short-fragment sequences into the genome frame to obtain the genome fine map, and then gap-closing the whole genome using the ABI3730XL sequencing platform. Gene prediction and COG annotation were performed according to a previous method [22,23].

### 2.4. Comparative Genomics

#### 2.4.1. Pan-Genome Analysis, Homologous Gene Analysis, and Phylogenomic Tree Inference

Pan-genome analysis was conducted using PGAP-1.2.1, which is a rapid and automatic pan-genomic pipeline for prokaryotes. The pathways of the core genes were identified using KEGG Mapper (https://www.genome.jp/kegg/mapper/search.html (accessed on 28 July 2022)). In addition, the whole genomes of 107 strains were included for homologous gene analysis using OrthoMCL with default parameters [24]. Thereafter, the orthologous genes were aligned using Mafft-7.313, and based on the alignments, a phylogenetic tree was created using the default algorithm [25]. Bootstrapping was performed with 1000 replicates.

#### 2.4.2. Tolerant and Non-Tolerant Strains Comparative Genomic Analysis

Tolerant strains with high growth rates and non-tolerant strains with low growth rates were selected for further analysis. The core gene set of tolerant strains was constructed using OrthoMCL [24]. Following a comparison of the whole genome of non-tolerant strains to this set, the results of the ortholog cluster were used to determine the genes unique to tolerant strains. Analogously, redundant genes were confirmed by contrasting the core gene set of non-tolerant strains with the whole genomes of tolerant strains.

### 2.5. Quantitative Real-Time PCR Analysis

*Lactobacilli* with the highest growth rates were incubated in MRS containing 1.2% bile salt for 12 h; the same growth conditions in fresh MRS were used as controls. Before RNA extraction from Gram-positive bacteria, 5 mL of culture was collected and 1 mL of a lysozyme solution (20 mg/mL) were added to the cell walls at 37 °C for 30 min. The cultures were collected by centrifugation before RNA extraction using TRIzol (Invitrogen, Carlsbad, CA, USA). cDNA was synthesized using the HiScript^®^ III 1st Strand cDNA Synthesis Kit (Vazyme, Nanjing, China) according to the manufacturer’s protocol. Quantitative real-time PCR (qPCR) was conducted in a total volume of 10 μL using Real-Time PCR Supermixes and Kits (Bio-Rad, Carlsbad, CA, USA). The primers were designed using Primer3.0, as shown in Table S2. 16S RNA was selected as the reference gene.

### 2.6. Plasmid Construction and Expression

Genomic DNA of *L. fermentum* 156 and *L. plantarum* S83 was isolated using the TIANamp Bacterial DNA Kit (Tiangen, Beijing, China). Plasmids were extracted using a TIANprep Mini Plasmid Kit (Tiangen, Beijing, China) according to the manufacturer’s instructions. Plasmid pSIP403 was digested using the restriction enzymes, NcoI and XhoI (Thermo Fisher Scientific, Shanghai, China). pSIP403-series primers were purchased from Sangon Biotechnology Laboratory (Shanghai, China), with the 5′ ends of the forward and reverse primers containing 22 bp homologous sequences, which were compatible with the end of the linearized vector. The PCR amplification of the target genes in genomic DNA was performed using a 2× Phanta Max Master Mix (Vazyme, Nanjing, China). All restriction enzymes digested DNA fragments (5.6kb NcoI–XhoI fragment), and the PCR products were purified with the GeneJET Gel Extraction Kit (Thermo Fisher Scientific, Shanghai, China) to obtain the linearized vector and target fragment; all products were constructed by homologous recombination using the ClonExpress Ultra One Step Cloning Kit (Vazyme, Nanjing, China). The correct transformants were verified by DNA sequencing, and the plasmids were isolated for electroporation. The preparation and electroporation of *L. plantarum* RS417 (non-tolerant strains) competent cells were conducted as described by Aukrust and Blom [26]. Stains harboring pSIP-derived plasmids were expressed as described by Nguyen et al. [27].

### 2.7. Development of a Method for the Rapid Screening of Bile Salt-Tolerant Lactobacilli

Primers were designed based on validated novel genes using Primer 3.0. Agarose gel electrophoresis was performed on the PCR amplification products of the 24 *lactobacilli*. Bile salt tolerance evaluation was performed to validate the PCR results, as described in Section 2.2.

### 2.8. Statistical Analysis

The data were analyzed using means and standard error (mean ± SEM). SPSS Inc. software (version 21.0; Chicago, IL, USA) was used to perform statistical analysis. The comparisons of differences between the means of the treatments were tested with a one-way ANOVA at a significance level of *p* < 0.05.

## 3. Results

### 3.1. Effect of Bile Salt on the Growth of 107 Lactobacilli

**Figure 1:**
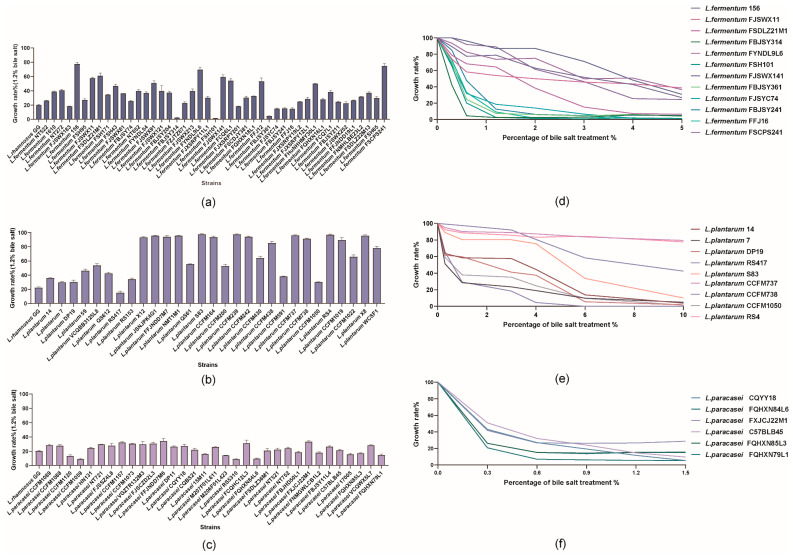
Growth rate of *lactobacilli* following 12 h incubations under bile salt conditions. (**a**–**c**) Growth rate of *L. fermentum*, *L. plantarum*, and *L. paracasei* in the presence of 1.2% bile salt, respectively. (**d**–**f**) Growth rate of *L. fermentum*, *L. plantarum*, and *L. paracasei* in the gradient bile salts, respectively. The experiments were performed in triplicate, and the results are expressed as mean ± SEM.

The 107 *lactobacilli* tested in this study showed high variability. In particular, 13% of the *lactobacilli* exhibited high tolerance (growth rate > 90%), 22% exhibited good tolerance (40% < growth rate < 90%), 62% exhibited low tolerance (5% < growth rate < 40%), and 3% did not grow. Of note, strains from the same species (particularly those from *L. fermentum* and *L. plantarum*) differed in this aspect, and these two species possessed strains that were both highly sensitive to and tolerant of 1.2% bile salt (Figure 1b). Some of the strains (*L. fermentum* FBJSY314, *L. fermentum* FSH101, and *L. fermentum* FBJSY361) were particularly sensitive to the action of bile salt, and they were mainly inhibited after 12 h (growth rate < 5%) (Figure 1a). For the studied *lactobacilli*, the growth rate of *L. paracasei* at the tested bile salt concentration (1.2%) was lower than 30% (Figure 1c).

An overall decreasing trend in bile salt tolerance was observed with increasing bile salt concentrations in the tested strains. *L. fermentum* 156 displayed excellent tolerance; however, the growth rate of most strains began to sharply decline at 3% and was below 40% at 5% (Figure 1d). Nevertheless, when the bile salt concentration increased from 0 to 10%, there were no significant differences in the growth rates of *L. plantarum* CCFM737 and RS4 within 12 h of incubation; however, the growth of RS417 was completely inhibited at 4% (Figure 1e). Notably, at the end of the treatment, when the bile salt concentration reached 1.5%, the reduction in the growth rate of *L. paracasei* was approximately 70% (Figure 1f).

### 3.2. Correlation Analysis of BSHs with Bile Salt Tolerance Phenotypes

**Figure 2:**
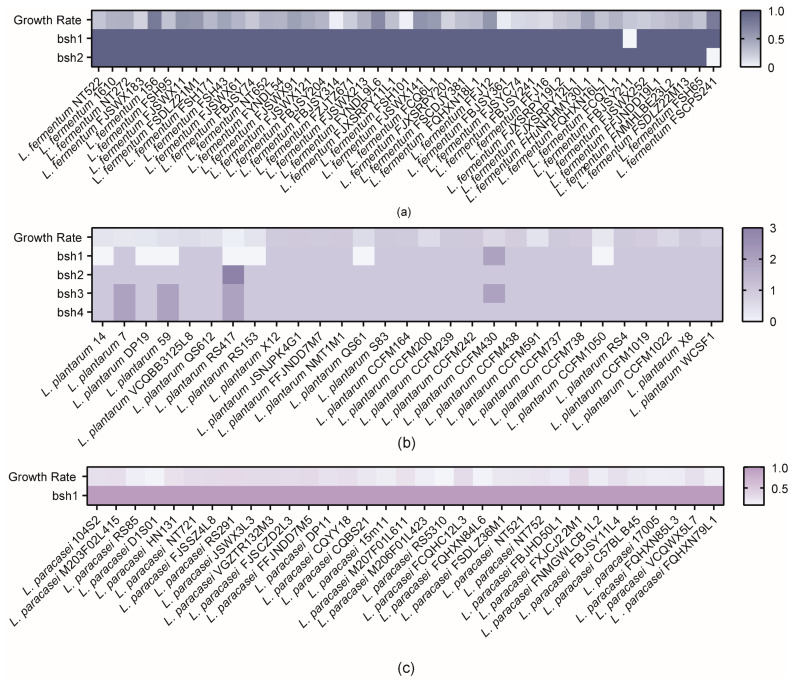
Heat map illustrating the distribution of BSH genes in *lactobacilli* and the growth rate of *lactobacilli* at 1.2% bile salt concentration. (**a**) *L. fermentum*, Spearman correction analysis, r = −0.07473. (**b**) *L. plantarum*, Spearman correction analysis, r = 0.07870. (**c**) *L. paracasei*, Spearman correction analysis, r = 0.

As shown in Figure 2, BSHs, which enable survival in bile salts, were widely distributed among the tested strains. However, in vitro experiments revealed that only a limited number of bacteria, mainly those belonging to *L. plantarum* and *L. fermentum*, could tolerate bile salts. Interestingly, *L. plantarum* RS417 had seven BSHs but showed poor bile salt tolerance, whereas *L. fermentum* FSCPS241, which lacked *bsh2*, had a good growth rate of 74.7%. Although all tested *L. paracasei* strains had BSHs, these strains failed to exhibit good tolerance. Furthermore, Spearman rank correlation analysis revealed a weak correlation between the presence of BSHs and the growth rate of the tested bacteria.

### 3.3. In Silico Analysis of Genome Features and Phylogenetic Analysis

**Figure 3:**
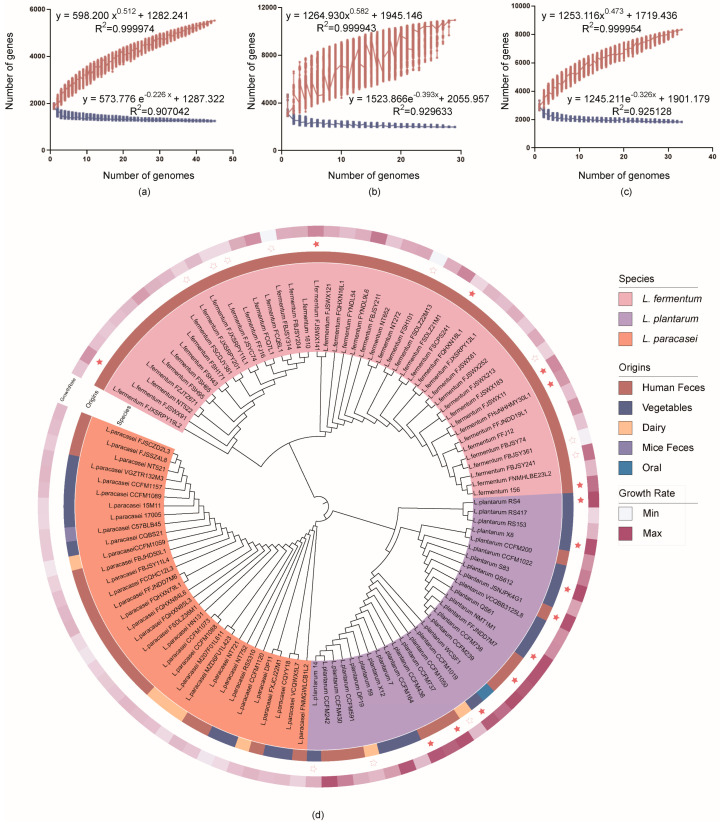
The pan-genome (in brown) and core-genome (in dark blue) estimates expressed as the accumulated number of genes in relation to the number of strains. (**a**) *L. fermentum*. (**b**) *L. plantarum*. (**c**) *L. paracasei*. (**d**) Phylogenetic tree of 107 *Lactobacillus* strains. Solid stars represent tolerant strains and hollow stars represent sensitive strains selected for comparative genomics.

The core genome of *L. fermentum* contained 1332 genes, that of *L. plantarum* contained 2117 genes, and that of *L. paracasei* contained 1941 genes (Figure 3a–c). This model describes the expansion of the pan-genome as a function of the number of genomes. The calculations approached the actual core genome and pan-genome sizes of the species when incorporating additional genomes, indicating the stabilization of the calculations of the core genome and pan-genome size.

For the differences in metabolic pathways among different species, in silico analysis revealed that the main functional genes of *L. fermentum* were enriched in amino acid transport and metabolism, while *L. plantarum* and *L. paracasei* were mainly focused on carbohydrate transport and metabolism. Although *L. paracasei* displayed an enrichment of genes in defense mechanisms, no effect on bile salt tolerance was found (Figure S1a). Furthermore, the enrichment of core genes by KEGG Mapper revealed that *L. paracasei* lacked genes related to arginine biosynthesis, riboflavin metabolism, and folate biosynthesis compared with other species (Figure S1b).

The evolutionary relationships between the 107 strains were assessed via a phylogenetic tree created using the 222 homologous genes shared by all strains. According to the clustering results, the bacteria of the three species were grouped into three clusters. Even within the same cluster, strains isolated from different sources were distributed on various evolutionary branches of the tree, indicating that the origin of the strains only agreed with their position on the phylogenomic tree to a low degree. Additionally, the bile salt-tolerant and non-tolerant strains did not clearly cluster in the adjacent branch. Although there was no significant correlation between strain origin and growth rate, strains from fermented vegetables that could grow after treatment and strains from oral cavities showed good tolerance. Strains from feces demonstrated various responses to this stress, with most surviving at 1.2% bile salt. In contrast, seven strains isolated from dairy products had a lower growth rate under stress (Figure 3d).

### 3.4. Comparative Genomics Analysis

#### 3.4.1. Potential Functional Genes Responsible for Bile Salt Tolerance

In this study, to identify novel functional genes associated with bile salt tolerance in *Lactobacillus*, six tolerant (>50% growth rate) and eight non-tolerant (<20% growth rate) strains of *L. fermentum* and nine tolerant (>90% growth rate) and three non-tolerant (<40% growth rate) strains of *L. plantarum* were selected for comparative genomics (Table S3). The analysis of *L. paracasei* was not considered because its growth rate in 1.2% bile salt was dissatisfactory.

**Figure 4:**
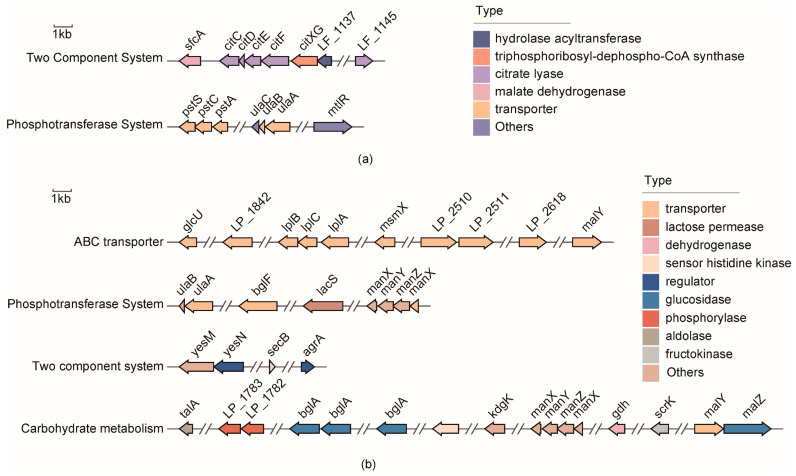
Gene clusters of the functional genes in bile salt-tolerant strains. (**a**) *L. fermentum* and (**b**) *L. plantarum*.

Twenty-five unique genes were present in the tolerant strains of *L. fermentum* (Table S3). Notably, these differentially expressed genes were enriched in two functional clusters, including eight associated with the two-component system and seven involved in the phosphotransferase system (PTS). In the former cluster, six genes belonged to the Cit B family of the two-component system involved in citrate and malate utilization. The latter cluster was enriched in phospho-ABC transporter proteins. Among them, the *ulaA* and *ulaB* genes associated with the PTS transporter subunits were responsible for lactose/cellobiose and ascorbic acid transmembrane transport, respectively (Figure 4a).

In the tolerant strains of *L. plantarum*, 87 unique genes were identified (Table S3). These differentially expressed genes were enriched in four functional clusters: ABC transporter, phosphotransferase system, two-component system, and carbohydrate metabolism (Figure 4b). The genes encoding transporter proteins are responsible for the transport of oligosaccharides and monosaccharides. Eight genes encoding PTS transport subunits were mainly involved in N-acetylglucosamine glucose and β-glucoside PTS. In particular, two genes encoding the two-component sensor, histidine kinase and DNA-binding protein response regulator, were responsible for sensing responses to environmental changes. Several genes encoding fructokinase, alpha-glucosidase, 6-phospho-beta-glucosidase, and maltose phosphorylase might be involved in carbohydrate metabolism to meet the energy requirements under bile salt stress.

#### 3.4.2. Potential Redundant Genes Responsible for Bile Salt Tolerance

Nineteen redundant genes were identified in *L. fermentum*. Combined with the annotations, these genes could be hypothesized to be involved in the valine, leucine, and isoleucine biosynthesis pathways. The same approach yielded 23 redundant genes in the *L. plantarum* strains. Based on the annotation results, these genes could be deduced to participate in DNA recombination, the restriction modification of DNA, and membrane integrity (Table S3).

### 3.5. Quantitative Real-Time PCR (qPCR) Assay for Detecting the Transcriptional Changes of Functional Genes

To assess the reliability of the results obtained by comparative genomics, genes with definite metabolic pathways (17 genes in *L. fermentum* and 27 genes in *L. plantarum*) were selected to determine their expression changes in *L. fermentum* 156 and *L. plantarum* S83, which were tolerant strains but had decreased growth rates after bile salt treatment (Table S3).

**Figure 5:**
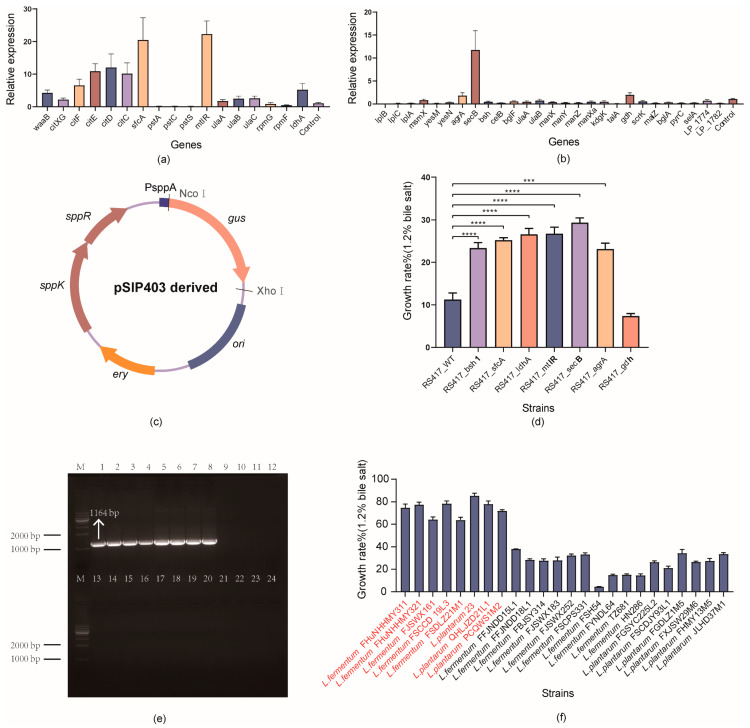
Validation of the functional genes by qPCR, overexpression in *lactobacilli*, and rapid screening of bile salt-resistant *lactobacilli* by novel target genes. (**a**,**b**) Relative expression of genes in *L. fermentum* 156 and *L. plantarum* S83, respectively. (**c**) pSIP403-series plasmid. (**d**) Growth rate of the induced strain in the presence of 1.2% bile salt; *** *p* < 0.001, **** *p* < 0.0001. (**e**) Rapid screening of tolerant strains by PCR amplification. (**f**) Growth rate of 24 *lactobacilli* under 1.2% bile salt treatment (strains from left to right correspond to lanes 1–24 in (**d**)).

Six genes (*citXG*, *citF*, *citE*, *citD*, *citC*, and *sfcA*) associated with the two-component system were upregulated by 2.19–20.48 fold in *L. fermentum* 156. Of the seven genes associated with the PTS system, only *mtlR*, *ulaA*, *ulaB*, and *ulaC* were upregulated following bile salt treatment, with *mtlR* being the most significantly upregulated gene (22.27 fold) (Figure 5a). Of the 27 specific genes in *L. plantarum*, only *agrA* and *secB* associated with the two-component system and *gdh* of the pentose phosphate pathway were upregulated by 1.8, 11.74, and 1.95 fold, respectively. Notably, in *L. plantarum*, the genes associated with the PTS system (*celB*, *bglF*, *ulaA*, *ulaB*, *manX*, *manY*, *manZ*, and *manX*) were downregulated. These results differed from the upregulation of the *ulaA* and *ulaB* genes in *L. fermentum* under the same treatment conditions, implying differences in bile salt tolerance mechanisms between the different species (Figure 5b).

### 3.6. Validation of the Functional Genes via Overexpression in Lactobacillus

New expression vectors were constructed based on the pSIP-vector series by exchanging the target genes with the *gus* (Figure 5c). The *sfcA*, *ldhA*, and *mtlR* genes encoding malate dehydrogenase, lactate dehydrogenase, and mannitol operon transcriptional antiterminator from *L. fermentum* and the *secB*, *agrA*, and *gdh* genes encoding preprotein translocase, DNA-binding response regulator, and glucose-1-dehydrogenase from *L. plantarum* were cloned into pSIP403, resulting in seven plasmids in which target gene transcription was controlled by the promoter PsppA, using *bsh1* as a reference gene. A significant increase of 11.86–18.08% in the growth rate was observed in the induced strains compared with that in the wild type, except for the *L. plantarum*-derived *gdh* gene, indicating a correlation between these genes and bile salt tolerance function (Figure 5d).

### 3.7. Sensitivity and Specificity of PCR Amplification Using the Developed Primer

In this study, the most significantly overexpressed gene (*sfcA*) was selected as a target for screening. PCR amplification was performed using 24 randomly selected *lactobacilli* based on the nucleotide sequence of *sfcA* (1164 bp), and eight strains were found to possess a single band around 1000 bp, indicating the presence of the *sfcA* gene in these strains (Figure 5e). The bile salt tolerance of 24 strains was also evaluated, and eight strains with the *sfcA* gene showed a good bile salt tolerance ability (growth rate > 60%) (Figure 5f).

## 4. Discussion

Bile contributes to the digestion and absorption of lipids, and it can further disrupt the phospholipids of the cell membrane and affect cell homeostasis through antimicrobial action [28]. BSHs are generally assumed to significantly contribute to the bile salt tolerance function of the bacteria encoding them. However, few studies have mined novel genes and evaluated their contribution to bacterial bile salt tolerance [29]. Based on the phenotypic differences of 107 *lactobacilli*, this study aimed to reveal the relationships between novel genes and bile salt tolerance function by combining comparative genomics and molecular biological methods. Consequently, we mined novel genes and used them as targets to rapidly screen for bile salt-tolerant *lactobacilli*.

Ox gall is normally selected as an alternative to human bile to evaluate the bile salt tolerance of bacteria in vitro owing to its similar composition. In the present study, numerous strains could cope with bile salt stress despite all three species displaying extensive intra-species variability in their growth rate. These results are consistent with those of other studies that found mutability in the bile salt response of strains isolated from different food products, including dairy [30,31], fermented starchy foods [19], and wine [21]. Human bile acid concentrations range from 8% in the gallbladder to 0.3–2% in the intestine [32]. Previous studies, which examined lower concentrations (0.25%, 0.5%, and 1%) than those discussed in this study, showed good bile salt resistance for all assayed strains and could therefore not obtain sensitive strains [33]. As a result, evaluating 1.2% bile salt would be more appropriate to obtain strains with extreme differences in tolerance ability. Although some researchers have argued that the distribution of strains tolerant and sensitive to bile salts is not species-specific [34], *L. plantarum* appears to exhibit a surprising tolerance ability [35]. *L. plantarum* WCSF1 could even grow during the application of 30% bile salt pressure [36]. In food applications, such as vegetable fermentation, *lactobacilli* are exposed to osmotic pressure when large quantities of salts or sugars are supplied; this could be an indication that as *L. plantarum* is usually isolated from fermented products in a high-osmolar environment, it better meets the prerequisites for bile salt tolerance in probiotics. In addition, in contrast to Reale et al. [6], who discovered that 116 genetically distinct strains of *L. paracasei* and *L. casei* isolated from food and feces could survive after incubation in 1.5% bile salt for 24 h, all *L. paracasei* tested in this study could not resume growth after treatment for 12 h with 1.2% bile salt. This variation in the bile salt tolerance ability of all strains was considerably affected by the varied experimental conditions used in the study (different types of media, bile salts, incubation time, and evaluation methods).

Owing to the complex and pleiotropic effects of BSHs, the bile salt-detoxifying effects of BSHs might be overestimated for BSH-positive strains. The presence of BSHs is often considered a desirable characteristic of probiotics, as the hydrolysis of BSHs is thought to reduce bile salt toxicity [37]. Accordingly, numerous researchers have tested this hypothesis, implying that BSHs may contribute to bile salt tolerance in *Lactobacillus*; however, the mechanism of protection is not fully understood [36]. Based on our findings, an absence of BSHs does not necessarily result in a reduction in bile salt tolerance. This result is consistent with that of Foley et al. [28], who reported that the expression of BSHs in vivo may result in a fitness cost owing to the increase in toxic deconjugated BAs. Therefore, despite the wide distribution of BSH genes, the ability to express this enzyme does not appear to correlate with the ability of *lactobacilli* to resist bile salt toxicity [38].

Here, phylogenomic analysis revealed that the further aggregation of the same species into evolutionary clusters did not clearly correspond to their isolation sources, indicating host speciation, similar to the case of *L. reuteri* and its niche speciation [39]. This notion is consistent with a previous perspective that *Lactobacillus* may be undergoing a reversion from a typical host-adapted lifestyle to that of a free-living species [40]. This evolutionary adaptation would facilitate the survival of strains of species in different ecosystems. Another explanation for the indistinguishability of the strains could involve fermentation, such as that in dairy products or fermented vegetables, being part of the human diet and being intimately connected to the human gastrointestinal tract. In this study, most strains isolated from fermented foods (e.g., fermented beverages, cheese, and fermented vegetables) showed excellent tolerance to bile salt exposure. These strains have adapted to growth under the hostile conditions imposed during the fermentation process, such as low pH, high osmolarity, and single nutrient proportions [6]. However, strains isolated from the same origin also showed significant strain specificity in their tolerance ability [30]. This result may be because the recorded isolation source of the tested species may not necessarily be where they evolved. Hence, bile salt-tolerant *lactobacilli* may be more easily obtained from fermented foods; however, the reliable validation of the bile salt tolerance of bacteria from their source alone is still required. The tolerance mechanisms or genes present in the bacteria appear to be better determinants of bile salt tolerance than the sources of the strains.

To illustrate the observed differences between *lactobacilli* in bile salt tolerance ability, potential gene markers were searched among the complete genome sequences. Owing to the ability of bile salts to disrupt the structure of cell membranes, they are usually completely ionized at physiological pH and retained in the outer half of the bilayer unless a transport system is present [3]. Thus, the active elimination of bile salts that accumulate in the cytoplasm is a common mechanism to resist bile salt toxicity. Several multidrug transporters (MDRs) have been described to mediate bile salt tolerance in *Lactobacillus* [41,42]. Furthermore, the reorganization of central metabolic pathways, particularly at the level of glycolysis, might be critical for bile salt tolerance [17,43]. Similarly, our findings revealed novel genes under this recombinant pathway that encode fructose-6-phosphate aldolase and glucose-1-dehydrogenase in the pentose phosphate pathway, fructokinase in the fructose and mannose metabolism pathway, alpha-glucosidase in galactose metabolism, and 6-phospho-beta-glucosidase and maltose phosphorylase in starch and sucrose metabolism. Based on these results, the reorganization of sugar metabolism after bile salt treatment increases energy production to counteract the detrimental effects of the bile salt threat. In addition, bile salt stress not only expands the utilization of carbon sources in *Lactobacillus* but also activates the PTS system, which mediates the uptake and phosphorylation of carbohydrates by phosphorylating various sugars and derivatives and then intracellularly transporting them via a phosphate cascade reaction [44]. As reported in *L. gasseri*, the cellobiose-specific EIIA transporter protein is upregulated in response to bile salt stress [17]. Analogously, our findings revealed the presence of novel genes encoding PTS transporters in *L. plantarum*. Finally, novel genes encoding citrate lyase and malate dehydrogenase, which play important roles in citric and malic acid utilization, were found to be associated with the two-component system [41]. Although the mechanisms involved in bile salt sensing and the response regulation of gene expression are not well-characterized, we hypothesized that *lactobacilli* use the two-component system to sense changes in the environment and establish new homeostasis under stress through changes in the abundance and activity of cellular proteins via signal transduction and the regulation of gene expression. In this regard, the two-component system and PTS systems might be metabolic pathways common to bile salt-tolerant *lactobacilli*.

The calculation of mRNA and protein expression levels is complementary, and both are critical for correlating genotypes and phenotypes [17]. The levels of upregulation associated with the uptake and utilization of external glucose, mannose, and fructose support the original view that for *lactobacilli* to survive bile salt stress, energy production mechanisms and transport systems must function more efficiently, allowing the bacteria to actively utilize extracellular sources of energy [45]. The upregulation of genes associated with the two-component system also demonstrated the role of bile salt perception in tolerance mechanisms [42]. In addition, gene expression systems used to express specific genes have promoted the confirmation of the crucial probiotic features of *Lactobacillus* [46]. However, *gdh* did not show good bile salt tolerance after expression and even reduced the survival rate of the wild-type strain in bile salt. This result may be related to host selection, where the vector with the target protein is toxic to the growth of the host, ultimately reducing the growth rate under bile salt stress [47].

The sensitivity of *lactobacilli* to bile salts was previously determined by measuring the growth of bacteria using the time delay method or survival rate [32]. The strains studied here were initially evaluated by calculating their growth rate from OD measurements. These approaches have proven to be reliable in assessing the ability of strains to grow in a given medium and have been commonly used in bile salt tolerance studies [48]. However, the growth of strains may be affected by different bile salt compositions and the interference of pH changes during bile salt tolerance [3]; this also explains the strain-specific behavior observed in previous studies in terms of bile salt tolerance. Thus, a rapid screening method based on specific targets avoids the negative effects of strains under growth conditions.

## 5. Conclusions

In this study, comparative genomics was employed to correlate functional genes and bile salt tolerance in *Lactobacillus*. This approach revealed the genes underlying the differences in bile salt tolerance ability despite the inclusion of members of the MRD family, which have been reported to be involved in bile salt tolerance. However, we also mined novel genes involved in the phosphotransferase system, the two-component system, and the carbohydrate metabolic pathway (which are associated with bile salt tolerance) for the first time. The construction of a pSIP-based expression system revealed that five genes are related to bile salt tolerance and that malate dehydrogenase (*sfcA*) can be used as a biomarker for screening bile salt-resistant *lactobacilli*. In conclusion, this study is a step closer to discovering marker genes for bile salt tolerance in *lactobacilli* and provides a new approach for the rapid screening of tolerant strains.

## Figures and Tables

**Table 1:** Bacteria and plasmids used in this work.

Bacteria and Plasmids	Relevant Characteristics
Strains	
*Lactobacillus fermentum* 156	Source of *bsh1*, *sfcA*, *ldhA*, and *mtlR* genes
*Lactobacillus plantarum* S83	Source of *secB*, *agrA*, and *gdh* genes
*Lactobacillus plantarum* RS417	Expression host
*Escherichia coli* DH5α	Cloning host
Plasmids	
pSIP403	spp-based expression vector with promoter PsppA

## Data Availability

All the strains sequences used in the article have been deposited in the NCBI database. Accessions numbers by strains are listed in Table S1. The 112 genes selected from two strains can be found in the BioProjects PRJNA658852 and PRJNA881784.

## Supplementary Materials

The following supporting information can be downloaded at: https://www.mdpi.com/article/10.3390/microorganisms10122371/s1, Figure S1: In silico analysis of the potential metabolic pathways. (**a**) Composition of the COG function categories in *lactobacilli*. (**b**) Metabolic pathway analysis of the homologous genes for different species through KEGG Mapper. Table S1: Detailed information of 107 *lactobacilli*; Table S2: Primer sequences; Table S3: Functional genes and redundant genes in *Lactobacillus* based on comparative genomic analysis.
