# Supplementary material for: Identification of Novel Bile Salt-Tolerant Genes in Lactobacillus Using Comparative Genomics and Its Application in the Rapid Screening of Tolerant Strains
Source: Microorganisms. 2022 Nov 30;10(12):2371. doi: 10.3390/microorganisms10122371 (PMC9786149; doi:10.3390/microorganisms10122371)
Supplement: Supplementary file 1 [file microorganisms-10-02371-s001.zip › microorganisms-2054213-supplementary.pdf]

Table S1. Detailed information of 107 *lactobacilli*.

Strains	Growth Rate%（Mean±SEM）	Origin	Size (Mb)	GC%	Accession No.
L. fermentum NT522	26.33±0.38	Human Feces	1.93	52.20	SRR21639374
L. fermentum 1610	38.84±0.75	Human Feces	1.97	52.26	SRR12560020
L. fermentum NT272	40.59±1.23	Human Feces	1.99	52.26	SRR12559831
L. fermentum FJSWX183	18.30±0.18	Human Feces	1.99	52.10	SRR12559549
L. fermentum 156	77.40±1.91	Human Feces	1.86	52.41	SRR12560021
L. fermentum FSH95	27.14±1.24	Human Feces	2.09	51.66	SRR12560122
L. fermentum FJSWX11	57.55±1.14	Human Feces	1.93	52.27	SRR12559785
L. fermentum FSDLZ21M1	61.01±3.20	Human Feces	1.98	52.11	SRR12559526
L. fermentum FSH171	34.46±0.60	Human Feces	2.06	51.85	SRR12559693
L. fermentum FSH43	46.41±2.28	Human Feces	1.95	52.34	SRR12560125
L. fermentum FJSWX61	36.24±0.08	Human Feces	1.97	52.13	SRR12559774
L. fermentum FBJSY74	25.48±1.05	Human Feces	1.93	52.17	SRR12559874
L. fermentum NT652	39.65±1.51	Human Feces	2.15	51.62	SRR12559826
L. fermentum FYNDL54	36.74±1.28	Human Feces	1.96	52.30	SRR12559673
L. fermentum FJSWX91	50.81±2.35	Human Feces	2.01	51.94	SRR12559763
L. fermentum FJSWX121	39.51±6.41	Human Feces	1.97	51.99	SRR12559553
L. fermentum FBJSY204	37.37±1.50	Human Feces	2.03	51.93	SRR12559863
L. fermentum FBJSY314	2.28±0.07	Human Feces	2.04	51.88	SRR12559463
L. fermentum FZJTZ671	22.89±1.28	Human Feces	2.01	51.92	SRR12559660
L. fermentum FJSWX213	39.67±2.28	Human Feces	1.92	52.29	SRR12559548
L. fermentum FYNDL9L6	69.64±2.51	Human Feces	1.93	52.24	SRR12559670
L. fermentum FJXSRPY11L1	30.16±1.77	Human Feces	2.05	51.86	SRR12559544
L. fermentum FSH101	1.61±0.06	Human Feces	2.06	51.93	SRR12559990
L. fermentum FJSWX141	59.44±2.37	Human Feces	1.96	52.21	SRR12559552
L. fermentum FCQ6L1	53.99±2.22	Human Feces	2.01	51.93	SRR12559742
L. fermentum FJXSRPY201	18.31±0.20	Human Feces	2.02	51.92	SRR12559541
L. fermentum FSCDJY381	30.45±1.56	Human Feces	1.96	52.31	SRR12559530
L. fermentum FQHXN18L1	32.63±0.11	Human Feces	1.94	52.14	SRR12559535
L. fermentum FFJ12	53.22±3.91	Human Feces	1.97	52.13	SRR12559984
L. fermentum FBJSY361	4.53±0.13	Human Feces	1.95	52.29	SRR12559753
L. fermentum FJSYC74	14.85±0.69	Human Feces	2.00	52.05	SRR12559546
L. fermentum FBJSY241	15.22±0.70	Human Feces	1.98	52.08	SRR12559475
L. fermentum FFJ16	14.62±1.20	Human Feces	2.00	51.97	SRR12559973
L. fermentum FJXSRPY19L2	24.66±0.26	Human Feces	1.97	52.03	SRR12559542
L. fermentum FJXSRPY12L1	28.47±1.60	Human Feces	1.97	52.18	SRR12559543
L. fermentum FHuNHHMY30L1	49.75±0.30	Human Feces	1.99	51.92	SRR12559494
L. fermentum FQHXN16L1	27.90±0.71	Human Feces	1.93	52.17	SRR12559536
L. fermentum FCQ7L1	38.36±1.61	Human Feces	2.03	51.99	SRR12559731
L. fermentum FBJSY211	24.41±0.71	Human Feces	1.94	52.03	SRR12559486
L. fermentum FJSWX252	22.4±1.75	Human Feces	2.02	51.89	SRR12559547
L. fermentum FFJNDD19L1	26.53±0.57	Human Feces	1.96	52.01	SRR12560042

L. fermentum FNMHLBE23L2	31.53±0.46	Human Feces	1.97	52.11	SRR12559540
L. fermentum FSDLZ22M13	36.95±1.25	Human Feces	1.95	52.21	SRR12559525
L. fermentum FSH65	29.71±1.58	Human Feces	2.04	51.79	SRR12560123
L. fermentum FSCPS241	74.70±2.75	Human Feces	1.89	52.28	SRR12559529
L. plantarum 14	35.77±0.79	Vegetables	3.10	44.73	SRR21639373
L. plantarum 7	29.87±1.06	Vegetables	5.61	46.79	SRR21639362
L. plantarum DP19	30.49±1.94	Dairy	3.30	44.51	SRR21639352
L. plantarum 59	46.19±1.85	Vegetables	5.19	47.39	SRR21639351
L. plantarum VCQBB3125L8	53.78±2.03	Vegetables	3.35	44.38	SRR12559741
L. plantarum QS612	42.46±1.34	Vegetables	3.35	44.37	SRR21639350
L. plantarum RS417	15.20±1.85	Vegetables	6.27	45.93	SRR21639349
L. plantarum RS153	34.18±1.29	Vegetables	3.78	46.08	SRR21639348
L. plantarum X12	93.27±0.76	Vegetables	3.34	44.34	SRR21639347
L. plantarum JSNJPK4G1	95.62±0.44	Human Feces	3.23	44.53	SRR12559477
L. plantarum FFJNDD7M7	93.99±1.21	Human Feces	3.23	44.53	SRR12559872
L. plantarum NMT1M1	95.56±0.55	Vegetables	3.30	44.43	SRR21639346
L. plantarum QS61	55.60±0.30	Vegetables	3.35	44.15	SRR12559707
L. plantarum S83	97.61±0.59	Vegetables	3.24	44.54	SRR12559465
L. plantarum CCFM164	93.53±1.32	Human Feces	3.26	44.49	SRR21639372
L. plantarum CCFM200	52.92±2.19	Human Feces	3.38	44.39	SRR21639371
L. plantarum CCFM239	97.45±0.49	Human Feces	3.13	44.66	SRR21639370
L. plantarum CCFM242	94.03±0.90	Human Feces	3.30	44.47	SRR21639369
L. plantarum CCFM430	64.16±1.98	Human Feces	3.27	44.47	SRR21639368
L. plantarum CCFM438	85.34±1.86	Human Feces	3.17	44.64	SRR21639367
L. plantarum CCFM591	38.17±0.39	Human Feces	3.42	44.25	SRR21639366
L. plantarum CCFM737	96.18±0.61	Human Feces	3.26	44.49	SRR21639365
L. plantarum CCFM738	91.34±0.72	Human Feces	3.23	44.52	SRR21639364
L. plantarum CCFM1050	30.17±0.66	Dairy	3.39	44.28	SRR21639363
L. plantarum RS4	97.03±0.67	Vegetables	3.26	44.49	SRR21639361
L. plantarum CCFM1019	89.29±3.13	Vegetables	3.35	44.39	SRR21639360
L. plantarum CCFM1022	65.73±2.21	Vegetables	3.40	44.31	SRR21639359
L. plantarum X8	95.63±1.05	Vegetables	3.38	44.39	SRR21639358
L. plantarum WCSF1	78.28±1.91	Oral	3.31	44.45	SRR8349598
L. paracasei CCFM1089	28.39±0.77	Vegetables	3.03	46.20	SRR12559706
L. paracasei CCFM1088	27.53±1.39	Dairy	3.15	46.15	SRR21639357
L. paracasei CCFM1120	13.21±1.23	Vegetables	3.07	46.31	SRR12559952
L. paracasei CCFM1059	8.77±1.03	Dairy	2.98	46.21	SRR21639356
L. paracasei HN131	24.22±1.04	Human Feces	3.01	46.39	SRR12560097
L. paracasei NT721	29.67±0.16	Human Feces	3.08	46.21	SRR12560091
L. paracasei FJSSZ4L8	27.93±2.41	Human Feces	2.98	46.32	SRR12559818
L. paracasei CCFM1157	32.18±1.17	Vegetables	3.08	46.30	SRR12559951
L. paracasei CCFM1073	30.55±0.62	Human Feces	3.05	46.24	SRR12560096
L. paracasei VGZTR132M3	29.78±3.22	Vegetables	3.06	46.35	SRR21639355
L. paracasei FJSCZD2L3	30.60±1.60	Human Feces	3.07	46.36	SRR12559820

L. paracasei FFJNDD7M6	34.23±2.83	Human Feces	2.84	46.37	SRR12559700
L. paracasei DP11	26.50±0.67	Dairy	3.02	46.28	SRR12559701
L. paracasei CQYY18	27.50±1.85	Vegetables	3.20	46.14	SRR12559703
L. paracasei CQBS21	21.85±1.85	Vegetables	3.10	46.30	SRR12559704
L. paracasei 15M11	16.07±0.50	Vegetables	2.74	46.54	SRR21639354
L. paracasei M207F01L611	25.80±0.60	Dairy	3.06	46.19	SRR12560093
L. paracasei M206F01L423	14.15±0.33	Dairy	2.77	46.49	SRR12560095
L. paracasei RS5310	8.78±0.58	Vegetables	3.18	46.19	SRR12559950
L. paracasei FCQHC12L3	31.34±3.46	Human Feces	3.06	46.16	SRR12559980
L. paracasei FQHXN84L6	9.58±0.62	Human Feces	3.00	46.31	SRR12559805
L. paracasei FSDLZ36M1	20.96±2.22	Human Feces	2.98	46.30	SRR12560118
L. paracasei NT521	22.20±1.67	Human Feces	2.97	46.28	SRR12560092
L. paracasei NT752	24.07±1.19	Human Feces	3.07	46.25	SRR12560090
L. paracasei FBJHD50L1	18.32±1.25	Human Feces	3.13	46.19	SRR12559988
L. paracasei FXJCJ22M1	33.24±1.18	Human Feces	3.38	45.98	SRR12560113
L. paracasei FNMGWLCB1L2	17.93±1.11	Human Feces	3.00	46.42	SRR12559816
L. paracasei FBJSY11L4	26.40±0.88	Human Feces	2.99	46.26	SRR12559986
L. paracasei C57BLB45	21.13±1.40	Mice Feces	3.10	46.30	SRR21639353
L. paracasei 17005	15.66±1.35	Vegetables	3.21	46.27	SRR12559705
L. paracasei FQHXN85L3	17.16±0.60	Human Feces	2.96	46.26	SRR12559804
L. paracasei VCQWX5L7	28.37±0.49	Vegetables	3.16	46.26	SRR12559945
L. paracasei FQHXN79L1	14.40±1.44	Human Feces	2.95	46.27	SRR12559807

Table S2. Primer sequences.

Genes	Primer	Sequences (5'-3')
reference	16S rRNA-F	TCCTACGGGAGGCAGCAGT
	16S rRNA-R	GGACTACCAGGGTATCTAATCCTGTT
waaB	LF156_0369-F	TGGAAACGGTGGGACAGA
	LF156_0369-R	CCAAAGCCGATTAGCAGG
citXG	LF156_1138-F	CCATTTTTTCGCTGGGTAT
	LF156_1138-R	CGGTCAGGGTTTGTTCTTG
citF	LF156_1139-F	GACTACCCGAACACCCCAG
	LF156_1139-R	AAGCGAGTTGCCCCACTAC
citE	LF156_1140-F	TAGCATCGCCGCCTTCG
	LF156_1140-R	ATGTGTTCCGTTTCCCG
citD	LF156_1141-F	CAAAATGGCATCCAAATC
	LF156_1141-R	AAGCACCCTTATCAACGG
citC	LF156_1142-F	TATGAACGCCAACCCCTT
	LF156_1142-R	GGATAGATCGCTGACCCC
sfcA	LF156_1144-F	AGCCAATACCCGAACCA
	LF156_1144-R	ACCAAGGAGGCCAGTCC
pstA	LF156_1200-F	CGTGACTAAGGTGATTCGGT
	LF156_1200-R	GCAGTTTCTGGTTAAGAGCG
pstC	LF156_1201-F	GCCACTCTGACTTCGATTTTA

	LF156_1201-R	GTTGTTCTACCCCCTTCTTGT
pstS	LF156_1202-F	CCGACCACAAGTCAGTTT
	LF156_1202-R	GCTTTTTAGCGTCAATCC
mtlR	LF156_1574-F	TTAGCAACATCAAGCACG
	LF156_1574-R	TTCAAATAAGTCACGGGT
ulaA	LF156_1571-F	GTTACTTACCATTACGCC
	LF156_1571-R	CTTGTTACCAAACCACTT
ulaB	LF156_1572-F	ATGGAGGAACAAACAATGAAG
	LF156_1572-R	TGGGTCCAAGAAGAAGTAATC
ulaC	LF156_1573-F	ATTGGTCCTGAAATTGCAC
	LF156_1573-R	AGATTGACGTCTTGGTTGG
rpmG	LF156_1608-F	AACAAGGCCAACACCCC
	LF156_1608-R	TCAACGAACCACACCCG
rpmF	LF156_1609-F	ACACCCTACCCCACCAC
	LF156_1609-R	TTACTTCACGGAACGCC
ldhA	LF156_1288-F	ATGGAGATGGTGCCCCT
	LF156_1288-R	ATCGTGATCCCGTTTTG
lplB	LPS83_2507-F	TAGGGGTTTGGAAAGGAA
	LPS83_2507-R	AACTGGTAGGATGTGTGG
lplC	LPS83_2506-F	TGTTGGGACCATAATGAACAG
	LPS83_2506-R	ACAGCATAGAAATCAAAGCGA
lplA	LPS83_2505-F	CGAAGAACTATGACGCAATGA
	LPS83_2505-R	AACCGCTACCAGAAGTAACCA
msmX	LPS83_2498-F	ATGTTGATTCTCGTGATTTT
	LPS83_2498-R	TTAGTGCGACTTACCTGTTT
yesM	LPS83_2502-F	CCAGAACTCGCCAATGT
	LPS83_2502-R	TGGGAATCTCAACCGCA
yesN	LPS83_2501-F	TCAATCACGCCAAAGACG
	LPS83_2501-R	TGACCCAGATACACCGCA
agrA	LPS83_1168-F	AGTTGGTGGTGGCGACA
	LPS83_1168-R	ACCCGGCCTGAGTTTGT
secB	LPS83_1081-F	GCTGGCGGAGAAGAAAA
	LPS83_1081-R	GCGACCGAACATAAGGG
bsh	LPS83_1788-F	TACCTGGAGACTTGTCCTCA
	LPS83_1788-R	ACTTCACATAGCCCTTTTTG
celB	LPS83_0388-F	TTTGGATTGGTGGAGCC
	LPS83_0388-R	CCATCATCATCGGAACG
bglF	LPS83_1775-F	GGACATGGGTTTATGGG
	LPS83_1775-R	AGCCTGCTGTTGACGAG
ulaA	LPS83_1792-F	TCACCCTTTGCTTCCATGT
	LPS83_1792-R	GCTGTTGTTGAGCCGTATT
ulaB	LPS83_1793-F	ATTCAGCAGATTACTTCTTTGTT
	LPS83_1793-R	TCTTGATCCAGAATTGTATTGAC
manX	LPS83_2131-F	TTAGTAAAAGAACCCGCTACA

	LPS83_2131-R	TAAGGCAACAAACTTCGTAAC
manY	LPS83_2130-F	CATTATTGTTGGCTTTGTGCT
	LPS83_2130-R	CTTGACTGACCTTTTTGGACT
manZ	LPS83_2129-F	GTTGGTGATACGCTTGT
	LPS83_2129-R	ACCTAATGGGTTTCCTT
manX	LPS83_2128-F	GGCGGGCAGTGTAAATCA
	LPS83_2128-R	GCCAACGCTAACGGAAGA
kdgK	LPS83_0382-F	GGCAGCACTCTATGAACAAT
	LPS83_0382-R	CTAAAACCAGCAAACCACTT
talA	LPS83_1791-F	AGTAGATGGGGTAACAACAA
	LPS83_1791-R	GAAACTGAGAATACAAGGGT
gdh	LPS83_3136-F	CGGTGACCTAGATGTTTGGG
	LPS83_3136-R	TATTGCCTGGCTTTTGATGC
scrK	LPS83_2500-F	TATTGGGGGTTACTCGC
	LPS83_2500-R	ATTGTCCCGCTGAAGGT
malZ	LPS83_1787-F	AACGCCACTACCTCAAAAG
	LPS83_1787-R	TGAAGACCCGATAACCATC
bglA	LPS83_1777-F	AATTCTACGAAACTTTCGC
	LPS83_1777-R	CTTTTTGTCATTAGCACCC
pyrC	LPS83_0385-F	AATGTTTATGCCTTGCTT
	LPS83_0385-R	TACTGATTTACTGACCCG
selA	LPS83_0384-F	CATTTACCGCTGGTGTTAG
	LPS83_0384-R	TAGTTCTGTTGTCGTGCCC
bglA	LPS83_1774-F	TAAAGACTTTTTGTGGGGCG
	LPS83_1774-R	ATTGACATATGCTTGCGATC
LP_1782	LPS83_1782-F	GAGATTCCTGGTGACTGG
	LPS83_1782-R	GTTCAAAATGATGGTGGT
**pSIP403-expression**		
	pSIP403-Em-F	ATCGTTTAACTTCAGGAGAGATTACATG
	pSIP403-Em-R	CGACTCATAGAATTATTTCCTCCCG
	pSIP403-tra-F	GCATAATAGTCTTGTAAGGTATTTAGCTGGCTG
	pSIP403-tra-R	TTTGAAGCTTGGCGCCTTCG
	pSIP403-F	CTCGAGGAATTCGGTACCCCG
	pSIP403-R	CCATGGGAATCATACTCCTATATATTATTTTATAAAGCATACTTTACG
	pSIP-*bsh*-F	TAGGAGTATGATTCCCATGGCAATGTGCACGAGCATCAACGTC
	pSIP-*bsh*-R	GGGGTACCGAATTCCTCGAGTTAATTTAATTCGACCGGCGCCC
	pSIP-156-1144-F	TAGGAGTATGATTCCCATGGCAATGCCTGAAAATGAAAACGATGAAGTTTTC
	pSIP-156-1144-R	GGGGTACCGAATTCCTCGAGTTAGTGTTTTTGCTGGGCAAAGTCG
	pSIP-156-1288-F	TAGGAGTATGATTCCCATGGCAATGTACAGTACCCACGAGCACG
	pSIP-156-1288-R	GGGGTACCGAATTCCTCGAGTTACTTAACGATGTTTTTAGTCCGTTGGCC
	pSIP-156-1574-F	TAGGAGTATGATTCCCATGGCAATGATTCGCTTTAGCAACATCAAGCAC
	pSIP-156-1574-R	GGGGTACCGAATTCCTCGAGTTAAGCTAGTTTTGAATCCATTAGAAGTTGGAGAAAC
	pSIP-S83-1788-F	TAGGAGTATGATTCCCATGGCAATGTGTACTGCCATAACTTATCAATCTTATAATAATTACTTCG
	pSIP-S83-1788-R	GGGGTACCGAATTCCTCGAGTTAGTTAACTGCATAGTATTGTGCTTCTGATCG

pSIP-S83-2665-F	TAGGAGTATGATTCCCATGGCAATGTGTACTAGTTTAACGATTCAAACCACGG
pSIP-S83-2665-R	GGGGTACCGAATTCCTCGAGTTAGTTTGCTAACCGGAACTGTTGGG
pSIP-S83-3136-F	TAGGAGTATGATTCCCATGGCAATGTATGAAGATTTAAATGGTAAGGTAGCAGTCATTAC
pSIP-S83-3136-R	GGGGTACCGAATTCCTCGAGTTATCCTTGACCATCCTTAAATGCAGGATAAAG
**Rapid screening**	
sfcA-F	AGCCTGAAAATGAAAACGATGAAGTTTTC
sfcA-R	TTAGTGTTTTTGCTGGGCAAAGTCG

Table S3. Functional genes and redundant genes in *Lactobacillus* based on comparative genomic analysis

Species	Group	Genes	Functions	Pathways
L. fermentum	Functional genes	LF_1137	hydrolase acyltransferase	
		LF_1152	glycosyl transferase	
		LF_0674	oxidoreductase	
		LF_0676	2,5-diketo-D-gluconic acid reductase	
		LF_1145	citrate lyase	
		LF_0369 (*waaB*)	UDP-D-galactose:(glucosyl) LPS alpha-1,6-D-galactosyltransferase	
		LF_1138 (*citXG*)	triphosphoribosyl-dephospho-CoA synthase	Two-Component System
		LF_1139 (*citF*)	citrate lyase subunit alpha	
		LF_1140 (*citE*)	citrate (pro-3S)-lyase subunit beta	
		LF_1141 (*citD*)	citrate lyase acyl carrier protein	
		LF_1142 (*citC*)	(citrate (pro-3S)-lyase ligase	
		LF_1144 (*sfcA*)	malate dehydrogenase	
		LF_1200 (*pstA*)	phosphate ABC superfamily ATP binding cassette transporter, membrane protein	Phosphotransferase system
		LF_1201 (*pstC*)	phosphate ABC transporter, permease PstC	
		LF_1202 (*pstS*)	phosphate ABC transporter substrate-binding protein	
		LF_1574 (*mtlR*)	mannitol operon transcriptional antiterminator	
		LF_1571 (*ulaA*)	PTS ascorbate transporter subunit IIC	
		LF_1572 (*ulaB*)	PTS system lactose cellobiose specific IIB component	
		LF_1573 (*ulaC*)	multidomain protein	
		LF_1608 (*rpmG*)	50S ribosomal protein L33	
		LF_1609 (*rpmF*)	50S ribosomal protein L32	
		LF_1288 (*ldhA*)	lactate dehydrogenase	
		LF_1293	hypothetical protein	
		LF_1803	hypothetical protein	
		LF_1830	hypothetical protein	
	Redundant genes	LF_1828	dihydroxy-acid dehydratase	
		LF_1829	hypothetical protein	
		LF_1830	sodium transporter	

		LF_1831	MFS transporter
		LF_1832	3-isopropylmalate dehydrogenase
		LF_1833	3-isopropylmalate dehydratase large subunit
		LF_1834	3-isopropylmalate dehydratase small subunit
		LF_1835	translation factor Sua5
		LF_1836	membrane protein
		LF_1837	hypothetical protein
		LF_1838	MFS transporter
		LF_1229	hypothetical protein
		LF_0712	membrane protein
		LF_1917	MFS transporter
		LF_1919	transcriptional regulator
		LF_1920	uracil phosphoribosyltransferase
		LF_1921	estradiol 17-beta-dehydrogenase
		LF_1922	molybdenum ABC transporter ATP-binding protein
		LF_1923	threonine dehydratase
L. plantarum	Functional genes	LP_1780	antitermination protein BlgG
		LP_1784	LacI family transcriptional regulator
		LP_2182	flagellar biosynthetic protein FlhB
		LP_1733	lactose permease
		LP_2917	peptidase M10
		LP_1835	malate dehydrogenase
		LP_2852	D-alanyl-D-alanine carboxypeptidase
		LP_1789	hydrolase
		LP_2822	DNA-damage-inducible protein J
		LP_2737	alpha-1 2-mannosidase
		LP_0093	membrane protein
		LP_1776	transcriptional antiterminator
		LP_2875	site-specific recombinase, phage integrase family
		LP_1830	glutamine amidotransferase
		LP_0383	2-dehydro-3-deoxyphosphooctonate aldolase
		LP_1838	cell surface protein
		LP_1839	2,5-diketo-D-gluconic acid reductase
		LP_1840	NAD(P)-dependent oxidoreductase
		LP_1841	bile-acid 7-alpha-dehydratase
		LP_1164	bacteriocin immunity protein
		LP_1166	bacteriocin immunity protein
		LP_3133	2,5-diketo-D-gluconic acid reductase
		LP_3134	DUF1211 domain-containing membrane protein
		LP_3138	oxidoreductase
		LP_3139	organophosphate reductase
		LP_2621	oxidoreductase
		LP_2620	oxidoreductase
		LP_2619	2,5-diketo-D-gluconic acid reductase

	LP_2617	oxidoreductase	
	LP_2430	glycosyltransferase	
	LP_2508	benzene 1,2-dioxygenase	
	LP_2513	transcriptional regulator, Fis family	
	LP_2512	GntR family transcriptional regulator	
	LP_2509	AraC family transcriptional regulator	
	LP_2738	GntR family transcriptional regulator	
	LP_2132	DegA family transcriptional regulator	
	LP_1831	transcription regulator, HxlR family protein	
	LP_0389	transcription regulator	
	LP_3140	LysR family transcriptional regulator	
	LP_1844	transcription regulator	
	LP_1786	MFS transporter	
	LP_2511	multidrug ABC transporter ATP-binding protein	
	LP_2510	multidrug ABC transporter ATP-binding protein	
	LP_2618	MFS transporter	
	LP_1842	MFS transporter	
	LP_3137	sugar transporter	
	LP_2507 (*lplB*)	sugar ABC transporter permease	ABC trasporter
	LP_2506 (*lplC*)	sugar ABC transporter permease	
	LP_2505 (*lplA*)	ABC transporter substrate-binding protein	
	LP_2498 (*msmX*)	sugar ABC transporter ATP-binding protein	
	LP_2502 (*yesM*)	two-component sensor histidine kinase	Two-Component System
	LP_2501 (*yesN*)	DNA-binding response regulator	
	LP_1168 (*agrA*)	DNA-binding response regulator	
	LP_1081 (*secB*)	preprotein translocase subunit SecB	
	LP_1788 (*bsh*)	choloylglycine hydrolase	Phosphotransferase system
	LP_0388 (*celB*)	PTS cellbiose transporter subunit IIC	
	LP_1775 (*bglF*)	PTS beta-glucoside transporter subunit EIIBCA	
	LP_1792 (*ulaA*)	PTS ascorbate transporter subunit IIC	
	LP_1793 (*ulaB*)	pts, eiib	
	LP_2131 (*manX*)	PTS system sorbose subfamily IIB component	
	LP_2130 (*manY*)	PTS N-acetylgalactosamine transporter subunit IIA	
	LP_2129 (*manZ*)	N-acetylgalactosamine permease IID component	
	LP_2128 (*manX*)	PTS N-acetylglucosamine transporter subunit IIBC	
	LP_0382 (*kdgK*)	2-keto-3-deoxygluconate kinase	
	LP_1791 (*talA*)	fructose-6-phosphate aldolase	
	LP_3136 (*gdh*)	glucose-1-dehydrogenase	
	LP_2500 (*scrK*)	fructokinase	
	LP_1787 (*malZ*)	alpha-glucosidase	
	LP_1777 (*bglA*)	6-phospho-beta-glucosidase	
	LP_0385 (*pyrC*)	dihydroorotase	

	LP_0384 (*selA*)	L-seryl-tRNA selenium transferase
	LP_2773	integral component of membrane
	LP_1774 (*bglA*)	6-phospho-beta-glucosidase
	LP_1782	maltose phosphorylase
Redundant genes	LP_2623	Integrase
	LP_3036	Integrase
	LP_3266	Integrase
	LP_2630	virulence protein
	LP_3230	virulence protein
	LP_2614	hypothetical protein
	LP_2462	alpha-galactosidase
	LP_1803	XRE family transcriptional regulator
	LP_3235	hypothetical protein
	LP_3037	DNA replication protein
	LP_3213	phage tail protein
	LP_3214	endonuclease
	LP_3268	terminase
	LP_3117	DNA-packaging protein
	LP_2270	hypothetical protein
	LP_0556	restriction endonuclease
	LP_2613	cell surface protein
	LP_0805	hypothetical protein
	LP_0346	hypothetical protein
	LP_1638	hypothetical protein
	LP_1694	bacteriocin immunity protein
	LP_0502	hypothetical protein
	LP_1176	hypothetical protein

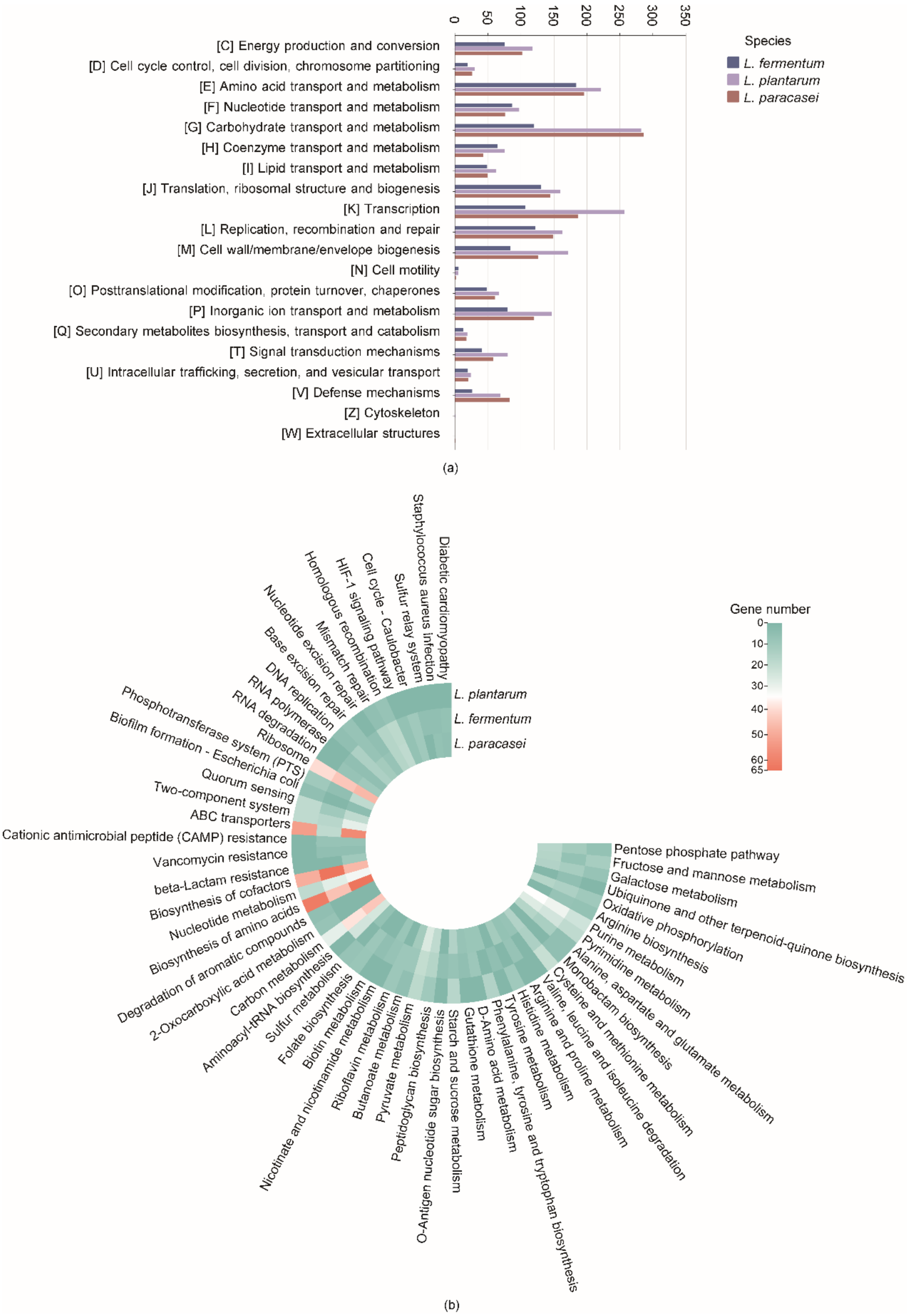

Figure. S1. In silico analysis of the potential metabolic pathways. (a) Composition of the COG function categories in *lactobacilli*. (b) Metabolic pathway analysis of the homologous genes for different species through KEGG Mapper.
